# Computational Fluid Dynamic Analysis of the Left Atrial Appendage to Predict Thrombosis Risk

**DOI:** 10.3389/fcvm.2018.00034

**Published:** 2018-04-04

**Authors:** Giorgia Maria Bosi, Andrew Cook, Rajan Rai, Leon J. Menezes, Silvia Schievano, Ryo Torii, Gaetano Burriesci

**Affiliations:** ^1^UCL Mechanical Engineering, University College London, London, United Kingdom; ^2^UCL Institute of Cardiovascular Science & Great Ormond Street Hospital for Children, London, United Kingdom; ^3^Biomedical Research Centre, NIHR University College London Hospitals, London, United Kingdom; ^4^Bioengineering Group, Ri.MED Foundation, Palermo, Italy

**Keywords:** Left Atrial Appendage (LAA), Atrial Fibrillation, CFD Simulation, Thrombosis Risk, LAA Morphologies

## Abstract

During Atrial Fibrillation (AF) more than 90% of the left atrial thrombi responsible for thromboembolic events originate in the left atrial appendage (LAA), a complex small sac protruding from the left atrium (LA). Current available treatments to prevent thromboembolic events are oral anticoagulation, surgical LAA exclusion, or percutaneous LAA occlusion. However, the mechanism behind thrombus formation in the LAA is poorly understood. The aim of this work is to analyse the hemodynamic behaviour in four typical LAA morphologies - “Chicken wing”, “Cactus”, “Windsock” and “Cauliflower” - to identify potential relationships between the different shapes and the risk of thrombotic events. Computerised tomography (CT) images from four patients with no LA pathology were segmented to derive the 3D anatomical shape of LAA and LA. Computational Fluid Dynamic (CFD) analyses based on the patient-specific anatomies were carried out imposing both healthy and AF flow conditions. Velocity and shear strain rate (SSR) were analysed for all cases. Residence time in the different LAA regions was estimated with a virtual contrast agent washing out. CFD results indicate that both velocity and SSR decrease along the LAA, from the ostium to the tip, at each instant in the cardiac cycle, thus making the LAA tip more prone to fluid stagnation, and therefore to thrombus formation. Velocity and SSR also decrease from normal to AF conditions. After four cardiac cycles, the lowest washout of contrast agent was observed for the Cauliflower morphology (3.27% of residual contrast in AF), and the highest for the Windsock (0.56% of residual contrast in AF). This suggests that the former is expected to be associated with a higher risk of thrombosis, in agreement with clinical reports in the literature. The presented computational models highlight the major role played by the LAA morphology on the hemodynamics, both in normal and AF conditions, revealing the potential support that numerical analyses can provide in the stratification of patients under risk of thrombus formation, towards personalised patient care.

## Introduction

Atrial fibrillation (AF) is the most common cardiac arrhythmia, affecting 1 to 2% of the population, and about 8% of individuals over 80 years of age (1). It is characterised by rapid and disorganised heart beating, triggered by electrical impulses usually originating in the roots of the pulmonary veins in the left atrium (LA), and has been identified as the leading cause of thromboembolic events, such as stroke and vascular dementia (2). As a result, AF brings about high social costs, around $26 billion per year in the US alone (3), with a projected increased incidence more than double by 2035 (4, 5). Hence, there is a pressing need for developing new effective strategies to predict and prevent thromboembolic events in patients suffering from AF (6).

It is estimated that more than 90% of left atrial thrombi responsible for thromboembolic events in AF forms in the left atrial appendage (LAA) (7, 8), a 2–4 cm long trabeculated finger-like projection from the main body of the LA. Despite active clinical research in the field, there is still ongoing debate about the function of this chamber, due to the difficulty to make direct measurements in this region of the heart.

The morphology of the LAA is largely variable from patient to patient, and is commonly classified into four different categories, on the basis of the shape it evokes: the “Chicken wing” morphology is the most common (48%), followed by “Cactus” (30%), “Windsock” (19%), and “Cauliflower” (3%) (9). However, the classification is often subjective and dependent on the imaging plane. In fact, the bidimensional nature of the imaging techniques commonly employed to observe the LAA (i.e., fluoroscopy and 2D echocardiography) can result into some overlapping between the different morphologies, as the distinguished shape, lobes, and branches depend on the angles of observation (10).

In normal sinus rhythm, the LAA acts as a highly contractile sac that obliterates its apex during atrial systole. Instead, in AF, its normal contractility reduces significantly, resulting in a decrease in echo Doppler velocities and dilation of the LAA. These conditions may promote the formation of hemostatic regions, leading to clotting (9).

The therapies currently available to reduce this risk include: (i) oral anticoagulation (i.e., warfarin) (8, 11, 12); (ii) LAA surgical exclusion (13); and (iii) LAA percutaneous occlusion (14, 15). Unfortunately, all these options present drawbacks that limit their application and efficacy, such as the risk of hemorrhagic consequences in the case of anticoagulation (8, 11, 12) and the complications associated with invasive and transcatheter procedures (e.g., vascular injury, air-embolic events and peri-device leaks) (13) for the surgical and percutaneous treatments, respectively (14, 15).

Therefore, it is crucial to develop methods able to identify the patient groups for which the risk of thromboembolic events justifies a therapeutic approach, and select the optimum therapy (6). Recent reports have suggested an association of the LAA morphology with the risk of clot formation, indicating the Chicken wing morphology as the least critical when compared to the non-Chicken wing shapes, although quantification of the risks are different in the literature, depending on the population examined (16). In particular, the Cauliflower shape appears to be associated with higher risk of thrombus development (17).

Few works in the literature present experimental and numerical studies analysing the flow patterns in the LA (18, 19), with some including the LAA in the model (20) and considering AF conditions (21–24). However, a direct comparison of different LAA morphologies with the onset of different hemodynamic parameters has not been produced yet.

In this light, the use of advanced computational simulations able to capture the fluid dynamic changes produced by AF might provide important insights into the phenomenon. The aim of this work is to develop computational fluid dynamic (CFD) models to investigate the relation between LAA shapes and the risk of thrombus formation, simulating normal sinus rhythm and AF conditions in 4 different patient-specific LAA morphologies.

## Materials and Methods

**Figure 1:**
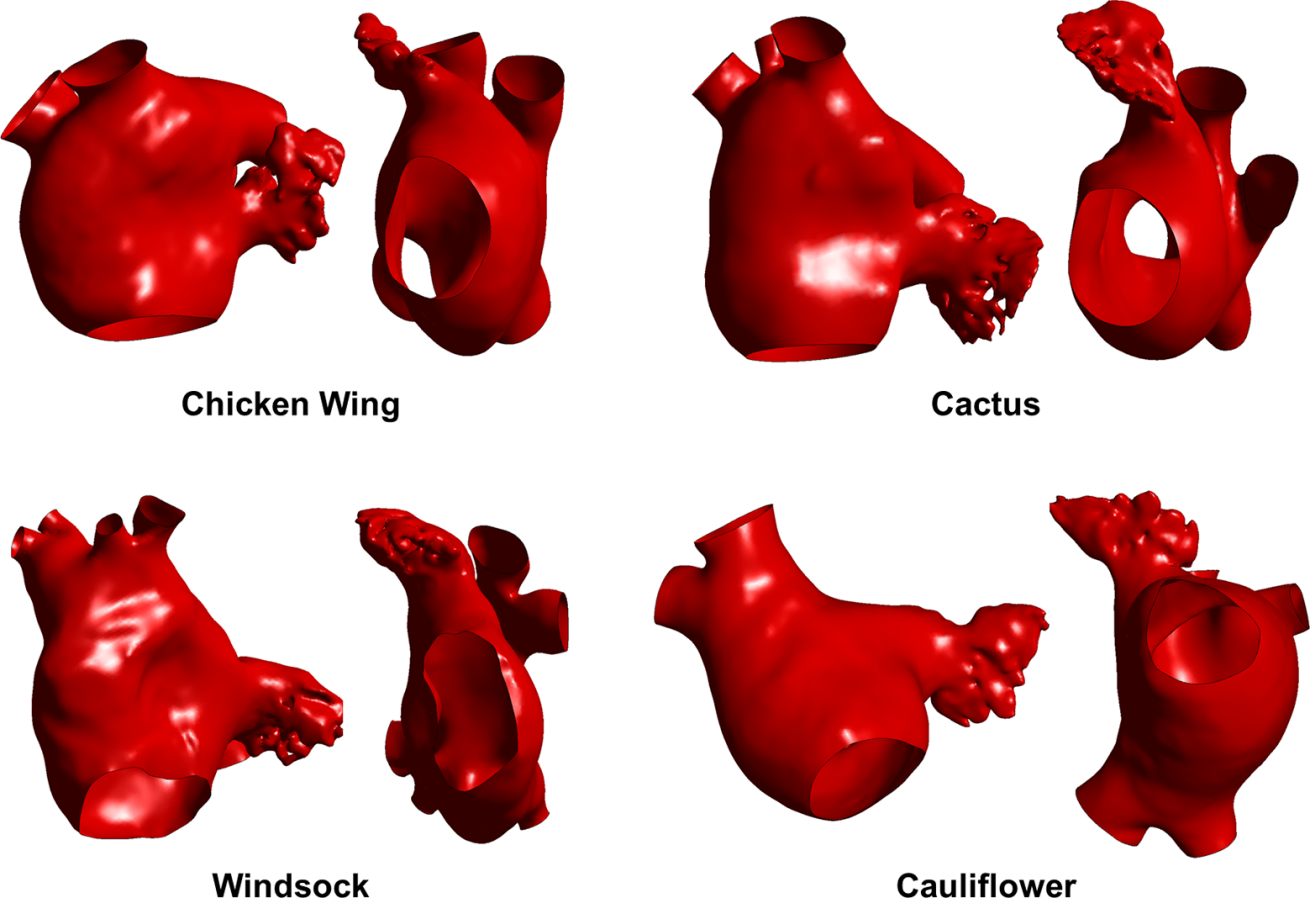
3D reconstruction of the four selected subjects represented in frontal and lateral view.

Computerised tomography (CT) images from adult patients who underwent a scan at University College London Hospital (London, UK) were used; all patients gave written informed consent. The patients were scanned for moderate coronary diseases but no known atrial pathology, hence were considered as control subjects. The images were segmented in the post-processing program Mimics (Materialise) to obtain the 3D anatomical shape of LAA and LA, following a common reconstructive method (25, 26). Four cases were selected by a cardiovascular morphology specialist as representative of the different morphological LAA classes (Figure 1); all selected shapes comprised the LA, the LAA, the pulmonary veins (PVs) and the mitral valve orifice. The number of PVs resulted variable, and only two of the four studied anatomies, the Chicken wing and Cauliflower models, had 4 standard vein openings in the LA (2 per each lung). The Cactus model and the Windsock presented 5 and 6 vein openings, respectively, due to changes in the right-side drainage patterns. These anatomical variations are reported to be common, and appear have some relation with AF onset (27).

In order to set up the computational fluid dynamic (CFD) models, all 3D anatomical models were meshed using Ansys ICEM CFD (Ansys, Inc.). Combination of tetrahedral and prism elements were used, with smaller tetrahedral elements in the LAA region and 5 layers of prism elements along the walls. After sensitivity analysis, the final mesh elements count was 2,227,544 for the Chicken wing case, 3,053,005 for the Cactus, 2,377,897 for the Windsock and 2,922,715 for the Cauliflower case. CFD analyses based on patient-specific anatomies were conducted using Ansys CFX (Ansys, Inc.) for all cases. Transient simulations were carried out over four complete cardiac cycles, with each cardiac cycle of 0.8 s duration (37.5% atrial diastole and 62.5% atrial systole), so as to allow the flow to fully develop. The differential equations were solved using a time step Δt = 0.5 ms.

**Figure 2:**
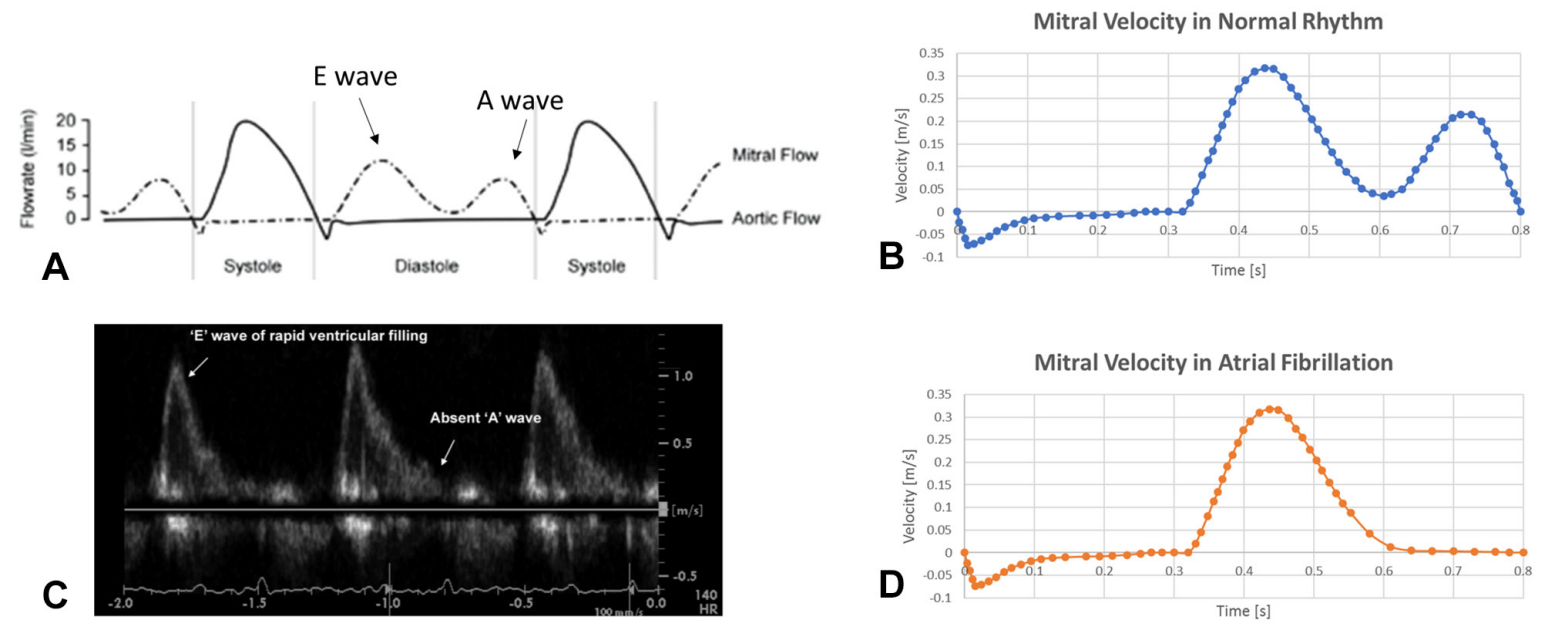
Boundary condition imposed in normal (top panel) and pathological (bottom panel) conditions. **(A)** flow rate across the mitral valve for a healthy generic subject (28): the atrial emptying phase (shown as ventricular diastole in the picture) is divided into E wave – representing the early atrial systole – and A wave – representing the atrial contraction. **(B)** Mitral valve velocity derived from the flow rate represented in **(A)** by dividing for the mitral valve orifice area, which was then imposed as boundary outlet condition. **(C)** Echocardiography study showing the lack of A wave in case of AF (29), as atrial contraction is almost absent. **(D)** Velocity profile derived for the mitral valve orifice in case of AF.

Rigid walls and no-slip condition were imposed at the LA and LAA surfaces, whilst open boundary condition was set at the pulmonary veins inlet, where constant pressure *P_pv_* equal to zero was imposed; and to the mitral valve outlet, where a different velocity waveform was imposed to simulate normal and AF conditions. For the healthy case, the flow rate across the mitral valve was derived from the international regulation ISO5840-1:2015 (28) and atrial emptying included both the first and second phase, represented by the *E-* and *A*-waves respectively (Figure 2A). Blood velocity across the mitral valve orifice was derived by dividing the instantaneous flowrate by the orifice area, thus reaching a peak of 0.32 m/s (Figure 2B).

For the AF case, the second atrial emptying wave (*A-*wave), was removed (29); as this is associated with atrial contraction, and is mostly absent in case of fibrillation (Figure 2C). The curve of the mitral valve velocity in the pathological condition was derived accordingly (Figure 2D).

As the Reynolds number, estimated on the basis of the peak mitral velocity and the maximum mitral valve diameter, was always below 3,000, a laminar model was chosen. The fluid was assumed incompressible and Newtonian, with density *ρ* and viscosity *μ* representative of normal blood (*ρ* = 1,060 kg/m^3^ and *μ* = 0.0035 Pa⋅s).

Blood flow patterns were characterised on the basis of the velocity and shear strain rate (SSR). Velocity streamlines of simulated flows were used to visually analyse the fluid dynamics profiles. Flow parameters were quantitatively estimated along a line running from the centre of the LAA ostium to the LAA tip, following the centre of gravity of each cross section. In addition, volume integrals of both velocity and SSR were calculated for all cases, to allow a global quantification of the fluid parameters in the entire LAA volumes.

A virtual contrast dye was included in the simulations, defined as an additional volumetric scalar variable, with the aim to estimate the residence time in different fluid regions and obtain qualitative data of the location more subjected to blood stagnation. Uniform density was assigned to the virtual contrast agent in the entire fluid domain at the first step in the analysis; this is progressively washed out during the 4 cardiac cycles. The remaining volume of contrast agent at the end of the simulations was normalised against the entire fluid domain volume, and was compared for all the cases.

## Results

CFD analyses depicted the flow dynamics produced in the four representative LAA morphologies. No significant cycle-to-cycle difference was observed after the second cardiac cycle, thus confirming that the flow became fully periodic after the first cycle.

The velocity values in the LAA were always substantially lower than in the LA, for all models, independently of the presence of AF and of the phase in the cardiac cycle.

**Figure 3:**
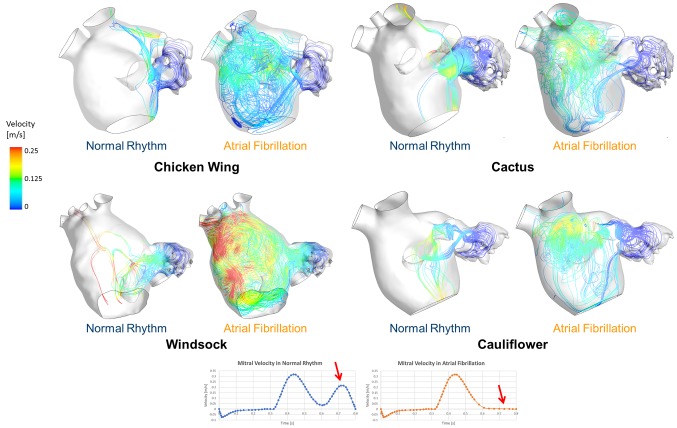
Instantaneous velocity streamlines passing through the LAA orifice represented for all LAA morphologies, taken at *t* = 0.75 s of the last simulated cardiac cycle, for both normal and pathological AF condition. This instant represents the second peak in the velocity profile, which is associated with the A-wave only in healthy conditions, as the A-wave is absent during AF.

At the peak of the *E*-wave, corresponding to the highest peak in the mitral flow velocity, the flow patterns in normal rhythm and AF appear very similar. As expected, differences become substantial during the late filling peak, when in normal conditions the mitral valve flow velocity reaches the second peak associated with the *A*-wave, which is absent in AF (Figure 3). Contrary to normal conditions, in the presence of AF all LAs show areas of low velocity recirculation (Figure 3). In particular, in AF case, the Chicken wing morphology presents the lowest LA velocities (v_max_ = 0.18 m/s), while the Windsock the highest (v_max_ = 0.38 m/s).

**Figure 4:**
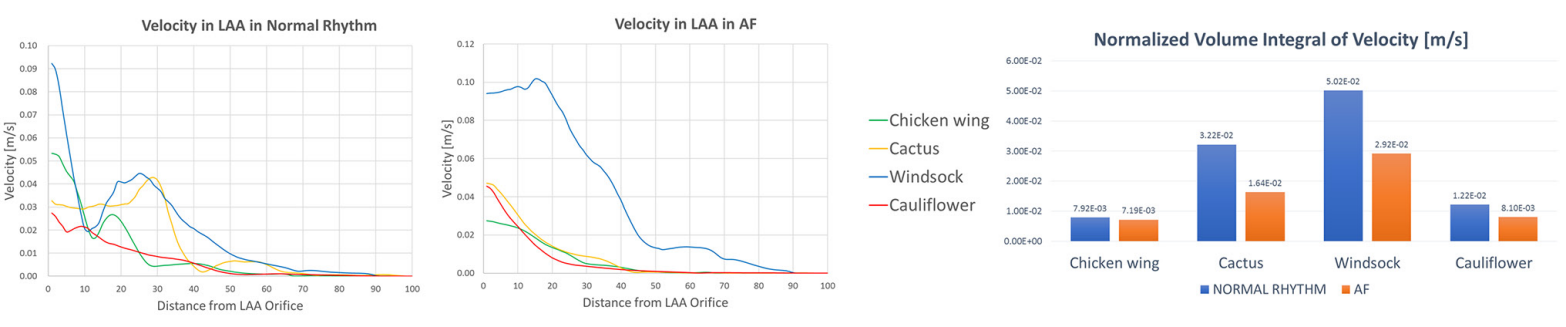
Velocity magnitude along the LAA, from the LAA orifice to the LAA tip, in normal (left panel) and AF (middle panel) conditions, for the different LAA shapes; velocity values are progressively decreasing from the LAA orifice to the LAA tip. In the right panel, during the same cardiac cycles phase, the volume integral of the velocity magnitude, normalized according to the different LAA volumes, decreases for all the LAA shapes when in AF conditions.

The velocity magnitude along the length of the LAA decreases from the orifice to the tip, thus making the LAA tip regions more subjected to fluid stasis, and therefore to a higher risk of thrombosis (Figure 4). The velocity was integrated spatially in the LAA volume, and normalised with respect to LAA volumes, in order to highlight differences between normal and pathological conditions. The resulting quantity indicates significant velocity reductions from normal to pathological conditions, with differences depending on the LAA morphology. The velocity reduction was highest for the Cactus shape (from 0.0322 to 0.0164 m/s) and smallest for the Chicken wing shape (from 0.00792 to 0.00719 m/s), while it changed from 0.0502 to 0.0292 m/s for the Windsock shape and from 0.0122 to 0.0081 m/s for the Cauliflower shape.

Comparing the 4 different LAA morphologies, the Chicken wing shape is characterised by the lowest velocity integral in normal conditions (0.00792 m/s against an average value of 0.02563 m/s for the 4 shapes), and in AF conditions (0.00719 m/s, against an average value of 0.01522 m/s).

**Figure 5:**
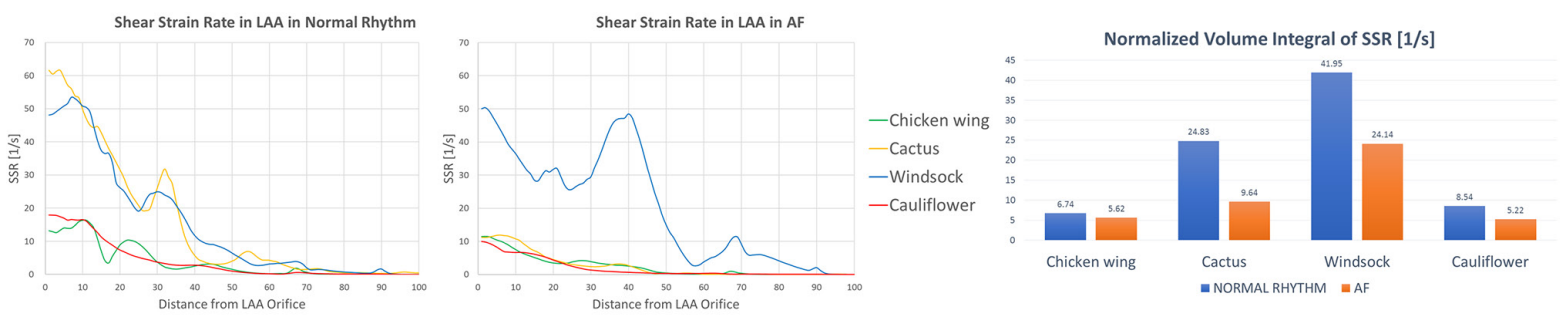
SSR along the LAA, from the LAA orifice to the LAA tip, in normal (left panel) and AF (middle panel) conditions, for the different LAA shapes; SSR values are progressively decreasing from the LAA orifice to the LAA tip. In the right panel, during the same cardiac cycles phase, the volume integral of the SSR, normalized according to the different LAA volumes, decreases for all the LAA shapes when in AF conditions.

SSR was calculated from the CFD results. Throughout the entire cardiac cycle, SSR values decrease from LAA ostium to the tip (Figure 5). Especially looking at the second phase of ventricular filling (*A*-wave), where the velocity waveform has the biggest difference from normal to AF condition. SSR at the centre of gravity of the LAA ostium in normal sinus rhythm was 13.2 s^−1^ for the Chicken wing, 61.6 s^−1^ for the Cactus, 50.1 s^−1^ for the Windsock and 17.9 s^−1^ for the Cauliflower; in AF condition, these values decreased to 11.4, 11.2, 48.1 and 9.9 s^−1^ respectively.

As for the velocity, SSR values were integrated spatially in the LAA volume. In all cases, the integral of the SSR, normalised with respect to LAA volumes, decreases from normal to pathological condition, and the amount of reduction depends substantially on the LAA shape: from 6.74 to 5.62 s^−1^ for the Chicken wing, from 24.83 to 9.64 s^−1^ for the Cactus, from 41.95 to 24.14 s^−1^ for the Windsock and from 8.54 to 5.22 s^−1^ for the Cauliflower.

The Chicken wing shape presents the lowest SSR value in normal condition, while the Cauliflower has the lowest SSR in AF condition.

**Figure 6:**
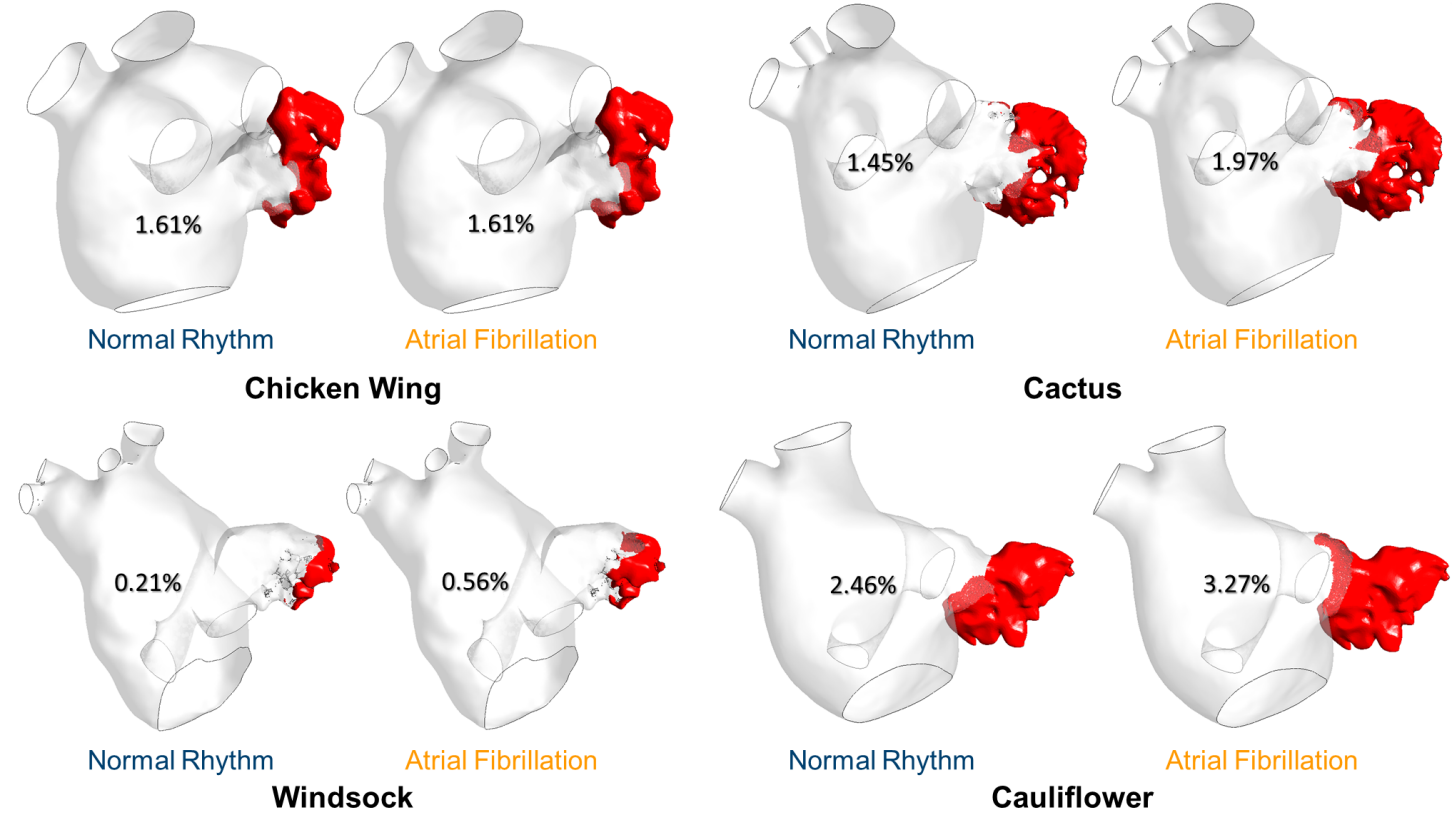
Residual [%] virtual contrast agent at the end of the CFD simulations for all the LAA morphologies and in both normal and pathological condition.

The virtual contrast dye simulations allow to visualize the regions where blood resides in the LAA for longer than one cardiac cycle. At the end of the 4^th^ cardiac cycle, the remaining dye agent was always located at the tip of the LAA. The normalised volume of residual contrast agent was equal to 1.61% for the Chicken wing for both normal and AF condition. In all other cases, this contrast agent volume was always higher in the AF case: 1.45 vs 1.97% for the Cactus, 0.21 vs 0.56% for the Windsock and 2.46 vs 3.27% for the Cauliflower (Figure 6).

## Discussion

In this work, CFD analyses were performed on 4 different LAA morphologies, representative of the different LAA classes typically identified by clinicians, in order to study the relationship between the LAA shape and the hemodynamics when imposing normal physiological flow and pathological AF conditions.

Computational modelling enabled a quantitative analysis of fluid-dynamic parameters, such as velocity streamlines and SSR values, that are impossible to measure non-invasively in clinics with sufficient accuracy. In fact, though transthoracic (TTE) and transoesophageal echocardiography (TEE) are available in the clinic to measure the blood flow velocity in both the LA and the LAA, both techniques can only provide average information on the velocity magnitude and direction in the sampling volume, without being able to give detailed spatial 3D information on the local flow conditions (30, 31). Ideally, 4D flow MR could provide similar information as the CFD analysis, but the spatial resolution, typically larger than 1 mm voxel, is not sufficient to assess a small region such as the LAA, and is thus not yet suitable for clinical applications (32).

Compared to the LA, lower velocity values were found in the LAA for all cases and in all conditions, thus making the LAA the region most subjected to fluid stagnation and, therefore, to potential thrombus development. Observation of the velocity streamlines departing from the LAA ostium during the second phase of atrial emptying, which in a normal subject corresponds to atrial contraction represented by the *A*-wave, shows the stronger contribution of the LAA region to the entire LA flow patterns in AF conditions, suggesting that if a thrombus forms in the LAA, this may be more likely to detach and travel towards the systemic circulation in the presence of AF.

Both flow velocity and SSR values at the LAA tip were always close to zero, thus making the LAA tip region the most dangerous in terms of thrombus development. Moreover, both velocity and SSR values decrease when AF flow is imposed, reflecting the pathological condition.

The volume integral of velocity and SSR provide an indication of the global contribution of these parameters in the LAAs. In the analysed cases, the Chicken wing shape has been found to be the one with lowest velocities overall; this might be due to the presence of cavities among the LAA lobes, that increase the tortuosity of the geometry and reduce the speed of the bulk flow. The Cauliflower morphology presented the lowest values of SSR in AF, thus confirming the clinical reports which classify this as the most prone to blood stagnation and, therefore, thrombus development (17).

Including the presence of a virtual contrast agent in the simulations allowed estimation not only its wash out in normal and pathological conditions, but also the location of the residual dye after 4 cardiac cycles. The LAA tip has been recognised as a flow stagnation area in both conditions and for all morphologies, probably due to the complexity of the LAA morphology. Moreover, during AF conditions, less blood was washed out, resulting in higher dye volume residing in the LAA chamber. The highest remaining contrast volume was found for the Cauliflower shape, again supporting the finding that the Cauliflower morphology can be associated with higher risk of thrombosis.

Amongst the four simulated cases, the Windsock shape resulted in the least likely to develop thrombus, being the one with highest velocities and SSR in both healthy and AF flow, and almost no residual dye volume at the end of the simulations.

These results might be affected not only by the morphology of the LAA, but also by the shape of the LA, which was different for every patient analysed, in terms of dimensions, structure and pulmonary venous drainage patterns.

### Limitations

All walls were considered rigid, not mimicking the real atrial contraction and movements during the cardiac cycle. This represent a major approximation, as the introduction of moving walls is likely to affect the flow in the LA by increasing the swirling motion, as reported in Chnafa et al. (33). However, this study is aimed at investigating the impact of the different LAA morphologies on the hemodynamics, rather than perfecting the model representation to each individual patient. Hence, the assumption allows to better isolate the potential effect of the appendage geometry, by abstracting it from the specific atrial motions experienced in each individual patient, especially in the case of rhythm disorders. Moreover, though motionless walls are not an accurate representation of the healthy condition, they are not far from the worst AF scenario (e.g., chronic AF), which is associated with limited atrial contraction.

No turbulence model was used in the analyses. Recent numerical reports suggest the presence of a transitional flow in the left atrium (33), predicting cycle-to-cycle variations in the flow field which occur for part of the diastole, at specific regions of the atrial chamber (depending on patient-specific anatomy and motions). In the presented study, the generalisation of laminar flow in the entire fluid domain and for the whole cycle duration allows substantial computational simplification, still providing a direct analysis of the effect of the morphology.

The fluid model was Newtonian, despite the blood having a shear thinning behaviour (i.e., its viscosity decreases with increasing shear stress). The effect of non-Newtonian properties of the fluid is likely to amplify the observed effects in terms of stagnation, as the viscosity in regions where SSR reduce below 100 s^−1^ can rise substantially, further reducing the estimated velocities.

Personalised inflow and outflow boundary conditions, including the effect of the dynamic mitral valve leaflets, might provide more realistic fluid dynamic results. However, the strategy taken in the present study was to limit the number of patient-specific factors other than the LA and LAA morphologies. Hence identical boundary conditions (i.e., pressure at the PVs and velocity profile at the mitral valve orifice) were used in all models, maintaining the focus of the investigation on the effect of the anatomical shape of the atrial chamber and its appendage on fluid dynamic parameters, such as velocity and SSR.

Lastly, though in the present study the Windsock shape appears to be associated with lower thrombotic risk, a much larger number of patient-specific cases is needed to clarify and generalise the relation between the LAA morphology and the hemodynamic behaviour leading to thrombus formation. More simulations will be performed in the future in order to achieve higher statistical significance and correlate our results with clinical data from AF patients with and without thromboembolic events development. These may lead to a new classification of the shape of appendage morphology, based on more objective quantitative measurements than currently adopted criteria, which remain far too subjective.

## Conclusions

The CFD models presented in this work enabled to highlight the effect of the LAA shape on the blood flow pattern and their variation in presence of AF. This computational study supports the hypothesis that the LAA morphology is a leading factor in the pathology, thus making some AF patients more at risk, depending on their LAA anatomical features. The presented approach, though in need of further improvement and large-scale validation, can become a powerful tool to quantitatively analyse parameters otherwise impossible to measure in clinics and to study the geometrical factors influencing thrombus formation. Lastly, this methodology has shown a significant potential in supporting clinical stratification of AF patients under high risk of thrombus formation, complementing the standard clinical information, thus supporting the selection of individualised therapies and improving the patient’s safety and standard of care.

## Ethics Statement

This study was carried out in accordance with the recommendations of the South East Research Ethics Research Committee, Ayelsford, Kent, UK, with written informed consent from all subjects. All subjects gave written informed consent in accordance with the Declaration of Helsinki. The protocol was approved by the South East Research Ethics Research Committee, Ayelsford, Kent, UK.

## Author Contributions

Each author was fully involved in the study and preparation of the manuscript, and has contributed significantly to the submitted work, in terms of conception and design of the study, analysis and interpretation of the results and critical review.

## Conflict of Interest Statement

The authors declare that the research was conducted in the absence of any commercial or financial relationships that could be construed as a potential conflict of interest.

## References

[1] Pellman J Sheikh F Atrial fibrillation: mechanisms, therapeutics, and future directions. *Compr Physiol* (2015) 5(2):649–65. 10.1002/cphy.c140047 25880508 PMC5240842

[2] Prince M Bryce R Albanese E Wimo A Ribeiro W Ferri CP The global prevalence of dementia: a systematic review and meta analysis. *Alzheimers Dement* (2013) 9(1):63–75. 10.1016/j.jalz.2012.11.007 23305823

[3] Kim MH Johnston SS Chu BC Dalal MR Schulman KL Estimation of total incremental health care costs in patients with atrial fibrillation in the United States. *Circ Cardiovasc Qual Outcomes* (2011) 4(3):313–20. 10.1161/CIRCOUTCOMES.110.958165 21540439

[4] Camm AJ Lip GYH de Caterina R Savelieva I Atar D Hohnloser SH 2012 focused update of the ESC Guidelines for the management of atrial fibrillation. *Eur Heart J* (2012) 33:2719–47. 22922413 10.1093/eurheartj/ehs253

[5] Naccarelli GV Varker H Lin J Schulman KL Increasing prevalence of atrial fibrillation and flutter in the United States. *Am J Cardiol* (2009) 104(11):1534–9. 10.1016/j.amjcard.2009.07.022 19932788

[6] Singh SM Micieli A Wijeysundera HC Economic evaluation of percutaneous left atrial appendage occlusion, dabigatran, and warfarin for stroke prevention in patients with nonvalvular atrial fibrillation. *Circulation* (2013) 127(24):2414–23. 10.1161/CIRCULATIONAHA.112.000920 23697908

[7] Al-Saady NM Obel OA Camm AJ Left atrial appendage: structure, function, and role in thromboembolism. *Heart* (1999) 82(5):547–54. 10.1136/hrt.82.5.547 10525506 PMC1760793

[8] Holmes DR Lakkireddy DR Whitlock RP Waksman R Mack MJ Left atrial appendage occlusion: opportunities and challenges. *J Am Coll Cardiol* (2014) 63(4):291–8. 10.1016/j.jacc.2013.08.1631 24076495

[9] Beigel R Wunderlich NC Ho SY Arsanjani R Siegel RJ The left atrial appendage: anatomy, function, and noninvasive evaluation. *JACC Cardiovasc Imaging* (2014) 7(12):1251–65. 10.1016/j.jcmg.2014.08.009 25496544

[10] Stöllberger C Ernst G Bonner E Finsterer J Slany J Left atrial appendage morphology: comparison of transesophageal images and postmortem casts. *Z Kardiol* (2003) 92(4):303–8. 10.1007/s00392-003-0903-x 12707789

[11] Hylek EM Evans-Molina C Shea C Henault LE Regan S Major hemorrhage and tolerability of warfarin in the first year of therapy among elderly patients with atrial fibrillation. *Circulation* (2007) 115(21):2689–96. 10.1161/CIRCULATIONAHA.106.653048 17515465

[12] Onalan O Lashevsky I Hamad A Crystal E Nonpharmacologic stroke prevention in atrial fibrillation. *Expert Rev Cardiovasc Ther* (2005) 3(4):619–33. 10.1586/14779072.3.4.619 16076273

[13] Ailawadi G Gerdisch MW Harvey RL Hooker RL Damiano RJ Salamon T Exclusion of the left atrial appendage with a novel device: early results of a multicenter trial. *J Thorac Cardiovasc Surg* (2011) 142(5):1002–9. 10.1016/j.jtcvs.2011.07.052 21906756

[14] Gary Gan CH Bhat A Davis L Denniss AR Percutaneous transcatheter left atrial appendage closure devices: role in the long-term management of atrial fibrillation. *Heart Lung Circ* (2014) 23(5):407–13. 10.1016/j.hlc.2013.12.008 24525145

[15] Lam YY Yan BP Doshi SK Li A Zhang D Kaya MG Preclinical evaluation of a new left atrial appendage occluder (Lifetech LAmbre™ device) in a canine model. *Int J Cardiol* (2013) 168(4):3996–4001. 10.1016/j.ijcard.2013.06.083 23871632

[16] Lupercio F Carlos Ruiz J Briceno DF Romero J Villablanca PA Berardi C Left atrial appendage morphology assessment for risk stratification of embolic stroke in patients with atrial fibrillation: a meta-analysis. *Hear Rhythm* (2016) 13(7):1402–9. 10.1016/j.hrthm.2016.03.042 27016474

[17] Di Biase L Santangeli P Anselmino M Mohanty P Salvetti I Gili S Does the left atrial appendage morphology correlate with the risk of stroke in patients with atrial fibrillation? Results from a multicenter study. *J Am Coll Cardiol* (2012) 60(6):531–8. 10.1016/j.jacc.2012.04.032 22858289

[18] Tanné D Bertrand E Kadem L Pibarot P Rieu R Assessment of left heart and pulmonary circulation flow dynamics by a new pulsed mock circulatory system. *Exp Fluids* (2010) 48(5):837–50. 10.1007/s00348-009-0771-x

[19] Vedula V George R Younes L Mittal R Hemodynamics in the left atrium and Its effect on ventricular flow patterns. *J Biomech Eng* (2015) 137(11):1–8. 10.1115/1.4031487 26329022

[20] Otani T Al-Issa A Pourmorteza A Mcveigh ER Wada S Ashikaga H A computational framework for personalized blood flow analysis in the human left atrium. *Ann Biomed Eng* (2016) 44(11):3284–94. 10.1007/s10439-016-1590-x 26968855

[21] Zhang LT Gay M Characterizing left atrial appendage functions in sinus rhythm and atrial fibrillation using computational models. *J Biomech* (2008) 41(11):2515–23. 10.1016/j.jbiomech.2008.05.012 18579148

[22] Koizumi R Funamoto K Hayase T Kanke Y Shibata M Shiraishi Y Numerical analysis of hemodynamic changes in the left atrium due to atrial fibrillation. *J Biomech* (2015) 48(3):472–8. 10.1016/j.jbiomech.2014.12.025 25547024

[23] Simmers MB Cole BK Ogletree ML Chen Z Xu Y Kong LJ Hemodynamics associated with atrial fibrillation directly alters thrombotic potential of endothelial cells. *Thromb Res* (2016) 143:34–9. 10.1016/j.thromres.2016.04.022 27179130

[24] Masci A Forti D Tommasi C Quarteroni A Functional Imaging and Modelling of the Heart. 9th International Conference, FIMH 2017; Toronto, ON, Canada (2017).

[25] Capelli C Bosi GM Cerri E Nordmeyer J Odenwald T Bonhoeffer P Patient-specific simulations of transcatheter aortic valve stent implantation. *Med Biol Eng Comput* (2012) 50(2):183–92. 10.1007/s11517-012-0864-1 22286953

[26] Bosi GM Capelli C Cheang MH Delahunty N Mullen M Taylor AM Population-specific material properties of the implantation site for transcatheter aortic valve replacement finite element simulations. *J Biomech* (2018). 10.1016/j.jbiomech.2018.02.017 PMC5889787 29482928

[27] Woźniak-Skowerska I Skowerski M Wnuk-Wojnar A Hoffmann A Nowak S Gola A Comparison of pulmonary veins anatomy in patients with and without atrial fibrillation: analysis by multislice tomography. *Int. J. Cardiol.* (2011) 146(2):181–5. 10.1016/j.ijcard.2009.06.047 19632731

[28] International Organization for Standardization. *ISO 4850-1:2015 Cardiovascular implants - cardiac valve prostheses part 1: general requirements*. Geneva, Switzerland: International Organization for Standardization (2015). p. 4850–1.

[29] Gautam S John RM Interatrial electrical dissociation after catheter-based ablation for atrial fibrillation and flutter. *Circ Arrhythm Electrophysiol* (2011) 4(4):e26–e28. 10.1161/CIRCEP.111.961920 21846876

[30] Fukuda N Shinohara H Sakabe K Onose Y Nada T Tamura Y Transthoracic Doppler echocardiographic measurement of left atrial appendage blood flow velocity: comparison with transoesophageal measurement. *Eur J Echocardiogr* (2003) 4(3):191–5. 10.1016/S1525-2167(02)00166-X 12928022

[31] Dentamaro I Vestito D Michelotto E de Santis D Ostuni V Cadeddu C Evaluation of left atrial appendage function and thrombi in patients with atrial fibrillation: from transthoracic to real time 3D transesophageal echocardiography. *Int J Cardiovasc Imaging* (2017) 33(4):0–498. 10.1007/s10554-016-1026-6 27853971

[32] Markl M Lee DC Furiasse N Carr M Foucar C Ng J Left atrial and left atrial appendage 4D blood flow dynamics in atrial fibrillation. *Circ Cardiovasc Imaging* (2016) 9(9):O90 10.1161/CIRCIMAGING.116.004984 PMC5019120 27613699

[33] Chnafa C Mendez S Nicoud F Image-based large-eddy simulation in a realistic left heart. *Comput Fluids* (2014) 94:173–87. 10.1016/j.compfluid.2014.01.030

